# Sex and gender differences in the molecular etiology of Parkinson’s disease: considerations for study design and data analysis

**DOI:** 10.1186/s13293-025-00692-w

**Published:** 2025-02-03

**Authors:** Samantha L. Schaffner, Kira N. Tosefsky, Amy M. Inskter, Silke Appel-Cresswell, Julia M. Schulze-Hentrich

**Affiliations:** 1 Pacific Parkinson’s Research Centre, Djavad Mowafaghian Centre for Brain Health, University of British Columbia, Vancouver, BC Canada; 2 Edwin S. H. Leong Centre for Healthy Aging, Faculty of Medicine, University of British Columbia, Vancouver, BC Canada; 3 Division of Neurology, Department of Medicine, University of British Columbia, Vancouver, BC Canada; 4 MD Undergraduate Program, University of British Columbia, Vancouver, BC Canada; 5 BC Children’s Hospital Research Institute, Vancouver, BC V5Z 4H4 Canada; 6 Department of Medical Genetics, University of British Columbia, Vancouver, BC V6H 3N1 Canada; 7 Department of Genetics/Epigenetics, Faculty NT, Saarland University, Campus, Building A2.4, 66123, 66041 Saarbrücken, Germany

**Keywords:** Parkinson’s disease, Genomics, Epigenomics, Transcriptomics, Study design, Data analysis

## Abstract

Parkinson’s disease (PD) is more prevalent in men than women, and presents with different clinical features in each sex. Despite widespread recognition of these differences, females are under-represented in clinical and experimental studies of PD, and much remains to be elucidated regarding the biological underpinnings of sex differences in PD. In this review, we summarize known contributors to sex differences in PD etiology across the life course, with a focus on neurological development and gene regulation. Sex differences that are established at conception and heightened during adolescence and midlife may partially embed future PD risk, due to the complex interactions between gonadal hormones, gene regulation, lifestyle factors, and aging. While the neuroprotective properties of estrogen are strongly implicated in reduced prevalence of PD in women, interactions with genotype and gender-biased lifestyle factors are incompletely understood. Consideration of sex and gender-related factors in study design, data analysis, and interpretation have the power to expedite our knowledge of the etiology of PD in men and in women, and to inform prevention and therapeutic strategies tailored to each sex.

## Abstract (plain language summary)

Parkinson’s disease (PD) more commonly affects men, and is known to have different symptoms in men and women. While this is in part due to the protective effects of estrogen in women, our understanding of why there is a sex difference in PD, and how it develops in each sex, is currently incomplete. This article provides an overview of factors throughout the lifespan that contribute to the differences between men and women in brain health and risk for PD, with a focus on hormones, gene regulation, and their intersections with lifestyle factors. We also discuss how researchers can consider sex and gender in future studies to enhance our understanding of how PD develops, and potentially develop sex-tailored prevention and treatment strategies.

## Highlights

- Genetic risk for PD is similar between men and women.
- Transcriptomic and epigenetic differences in men and women with PD have been reported, particularly in substantia nigra tissue.
- Emerging evidence suggests interactions between gene regulation, sex hormones, and lifestyle factors contribute to disease pathogenesis in each sex.
- Statistical approaches can be used to balance sex ratios and explore sex as a contributor to PD etiology, rather than a confounder.
- Increasing representation of women in PD clinical studies is a priority for future research endeavors.

## Background

Parkinson’s disease (PD) is the second most common neurodegenerative disorder, with prevalence increasing by 156% between 1990 and 2019, and 22 million individuals worldwide projected to be affected by 2050 [1–3]. Approximately 1.4 times more men than women were reported to be living with PD between 1990 and 2016, although more recent analyses have suggested that sex differences in prevalence may be narrowing over time [2–4]. As the biological underpinnings of PD and their interactions with environmental and lifestyle risk factors are becoming more clearly elucidated, insights into personalized risk profiles and intervention strategies are increasing [5–7].

One key area for development of future research studies, diagnostics, and treatments for PD is understanding the differences in its manifestation in men and women, and to which degree these differences are influenced by sex (biological and physiological characteristics) and/or gender (social constructs and identity). Epidemiological and clinical studies of PD focused on its expression in cisgender men and women capture both sex- and gender-related factors. For instance, both prevalence and clinical expression of PD differ between sexes: females with PD are more likely to present with tremor and experience depression, while males with PD are at greater risk for cognitive decline and experience more rigidity [8, 9]. This suggests potential sex-related biological underpinnings of PD and/or contributions of gender-related sociocultural and lifestyle factors to disease risk [10, 11].

While several articles have reviewed known sex- and gender-related considerations in PD to date, the use of sex and gender as variables in primary research studies assessing the molecular etiology of PD is only just beginning [8–11]. Case-control studies may be imbalanced by sex, and sex is treated inconsistently across studies, frequently adjusted for as a confounder rather than investigated as a variable of interest, interacting variable, or contributor to main effects [12, 13]. This can lead to incorrect conclusions if it is assumed that the relationship between predictor and outcome is the same between sexes [14]. The increasing number of large consortia recruiting thousands of cases and controls, the growing availability of public data for secondary analyses, and rapidly evolving technologies present prime opportunities for the analysis of sex-related molecular underpinnings of PD and their interactions with gender (when identity-related information is collected).

In this review, we summarize known and potential contributions of sex to PD risk in the context of genomics, epigenomics, and transcriptomics, provide suggestions to enhance representation of both sexes in clinical cohorts, and present guidelines for addressing sex as a contributor to PD risk in statistical analyses. We also identify current gaps and opportunities for understanding the interactions between sex and gender, genotype, and environmental factors in the context of PD etiology. Finally, we highlight the potential for PD prevention and intervention strategies targeted to each sex. For clarity, “males” and “females” will be used in reference to biological sex, while “men” and “women” will be used when referring to gender and when reviewing studies where the distinction between sex and gender is unclear.

## The role of biological sex in Parkinson’s disease susceptibility

### Gonadal hormones

Biological sex sets the stage for structural and functional differences in mammalian brain development which may influence later-life neurological disorder susceptibility. Sexual differentiation of the brain is first established weeks after conception with transcription of *SRY* from the Y chromosome in males, leading to formation of the testes and production of testosterone and other androgens [15]. Testosterone is converted to estradiol in the limbic system, which can shape astrocyte morphology, stimulate glutamate release from astrocytes and neurons, and modulate cell survival and death [16]. This surge of testosterone in early development establishes the first male-female differences in brain structure and connectivity, which program sensitivity to further hormone surges during adolescence, a phenomenon known as the “organization-activation” hypothesis [17, 18]. Both sexes also experience a decline in gonadal hormone levels at midlife [19, 20]. Taken together, fluctuations in gonadal hormone levels throughout the life course affect human brain structure, function, and behaviours distinctly in males and females, and the development of PD in each sex should be considered within this context. Importantly, the pre-diagnostic period for PD typically occurs during midlife and may include individuals experiencing prodromal PD symptoms for up to 10 years preceding motor symptom onset [21]. Understanding the impact of declining estrogen and testosterone levels on brain health during middle age is key to elucidating sex differences in disease susceptibility.

Human population studies during the midlife period as well as animal experiments point to a neuroprotective effect of estrogen in the context of aging and neurodegeneration. Age of menopause in women correlates strongly with the age of onset of PD, suggesting the 10-fold drop in estradiol levels that occur during the menopausal transition could relate to disease susceptibility [22]. Additionally, women who either have a later onset of menopause or use post-menopausal hormone therapy each have a reduced risk for PD [23, 24]. Estrogen therapy administered early during the disease course is also associated with a reduction in PD symptom severity in women, as measured by total Unified Parkinson’s Disease Rating Scale (UPDRS) score [25]. Several mechanisms for this estrogen-mediated neuroprotection have been proposed. Firstly, activation of the MAPK/ERK and PI3K/Akt pathways by estrogen protects against toxicity from glutamate, amyloid beta, and hydrogen peroxide in cultured neurons [26, 27]. Rodent studies also show a reduction in toxin-associated dopaminergic neuron loss in females compared with males and in estrogen-treated animals, mediated by down-regulation of pro-apoptotic proteins and up-regulation of anti-apoptotic proteins [28–30]. Although these experimental models cannot fully recapitulate the disease process in the human brain, they suggest that estrogen and its derivatives may shield against oxidative stress and neuron loss in mammals.

The role of androgens in PD susceptibility is less clear [31]. While androgens are aromatized to estradiol in both sexes, this is the primary mechanism of estradiol synthesis in males, and results in less circulating total estradiol compared with females who also synthesize this hormone from the ovaries [32]. Although increased PD susceptibility in males may be partly due to lower levels of converted estradiol, several groups have also investigated whether androgens are directly involved. Two studies reported lower plasma testosterone levels in men with PD compared to men without PD (50 and 68 individuals assessed for testosterone, respectively) [33, 34]. While a retrospective analysis in five men receiving testosterone replacement therapy showed an improvement in PD nonmotor symptoms, an eight-week testosterone intervention in 15 men with PD yielded no improvement in motor or nonmotor symptoms [34, 35]. A similar association of lower testosterone levels with Alzheimer’s disease, cognitive decline, and dementia has been reported, which may relate to the ability of testosterone to protect against β-amyloid accumulation in the brains of rodents and in cell culture experiments [19, 36, 37]. However, testosterone-based interventions in humans have yielded inconsistent results [19]. The effects of testosterone on neuroprotection in rodent models of PD are also unclear. Castrated rats experienced less neurodegeneration, oxidative stress, and motor deficits upon 6-OHDA treatment than intact rats, while castrated mice had impaired motor activity, glial activation, and striatal dopamine levels compared with intact mice [38–40]. Taken together, while a large body of research supports a neuroprotective effect of estrogens, there is insufficient evidence to conclude that androgens either worsen or improve risk for PD or its symptoms. This is consistent with the greater susceptibility to PD in men compared with women, suggesting the reduced estradiol levels from conversion of androgens may play a role in sex differences in PD.

### Gene regulation

Aside from gonadal hormones, gene expression and epigenetic regulation also shape sexual differentiation in the nervous system and may impact later life risk for PD. Random X-chromosome inactivation in females results in within-individual mosaicism, and genes that escape from X-inactivation are expressed biallelically, and often at an increased X-linked gene dosage in females [41]. Additionally, genomic imprinting, where either the maternal or paternal allele of a gene is epigenetically silenced, results in parent-of-origin-specific gene expression patterns [42]. Many autosomal loci also have sex-specific gene expression patterns, modulated by hormonal and physiological differences between sexes, differences in lifestyle/exposure between sexes, epistasis with the X and/or Y chromosomes, and differing magnitudes of pathway activation between sexes (“amplification”) [10, 43, 44]. For instance, the APOE ε4 allele is associated with increased neurofibrillary tangles in females compared with males, which may explain why female APOE ε4 carriers are at higher risk of developing Alzheimer’s disease than male carriers [45]. However, conflicting results have been reported with respect to sex differences in autosomal genetic risk for PD (Table 1). A 2021 study in > 200,000 individuals reported a high correlation between genome-wide association study (GWAS) analyses for PD in males and females, and similar heritability for PD in each sex [46]. Another recent study found overlap between PD GWAS risk loci and single nucleotide polymorphisms (SNPs) associated with age at menarche and/or age at menopause, while a third study in a Korean population reported female-specific associations of five SNPs with PD [47, 48]. These results suggest that while overall genetic risk for PD is similar between males and females, there may be an interaction between genes and hormonal and/or sociocultural factors that contribute to the differing incidence and presentation of PD between sexes.

**Table 1:** Sex- and gender-stratified studies of genomics, transcriptomics, and epigenomics of Parkinson’s disease

Type	Platform	Sample	Findings	Reference
Genomics; Transcriptomics	Microarray (various); RNA-seq	GWAS summary statistics from PD (13 708 cases, 95 282 controls), age at menarche (368 888 females), age at menopause (69 360 females) Post-mortem frontal cortex: 12 male control vs. 11 female control, 12 male PD vs. 7 female PD	7 loci associated with PD and age at menarche (FDR < 0.05), including 5 sex-specific eQTLs 4 loci associated with PD and age at menopause (FDR < 0.05), including 1 sex-specific eQTL Lack of sex-associated differential expression in PD post-mortem frontal cortex	[47]
Genomics	Korean Chip (K-CHIP) microarray	2390 male control, 496 male PD, 2610 female control, 554 female PD	5 SNPs associated with PD in female-only analysis (1 *LRRK2*, 4 *SNCA*; *P* < 1.03 × 10^–7^) No significant associations in male-only analysis	[48]
Genomics	Microarray (various)	89 660 male control, 13 020 male PD, 90 662 female control, 7947 female PD	No sex-specific associations with PD High correlation between male and female PD GWAS (r_g_=0.877) Similar heritability between male (*h*^2^ = 0.21) and female (*h*^2^ = 0.19) PD cases	[46]
Transcriptomics meta-analysis	Microarray (various) and RNA-seq	Substantia nigra tissue (193, 52% PD, 37% female); dopaminergic neurons (49, 55% PD, 45% female); iPSC-derived dopaminergic neurons (33, 52% PD, 33% female); single-cell substantia nigra data (43, 48% PD, 56% female)	At FDR < 0.05: 36 female-specific genes 539 male-specific genes 37 sex-dimorphic genes	[55]
Transcriptomics meta-analysis	Microarray (various)	Frontal cortex tissue (73, 49% PD, 47% female), striatum tissue (85, 47% PD, 41% female), and substantia nigra tissue (109, 54% PD, 37% female)	237 DEGs in substantia nigra (FDR < 0.05), 75 with increased expression in males and 162 with increased expression in females 15 genes with sex- and PD-specific expression across all 3 brain regions	[54]
Transcriptomics meta-analysis	Microarray (various)	Substantia nigra tissue (73 male control, 67 male PD, 39 female control, 48 female PD)	9 chromosomal segments each differentially expressed between male and female controls, and male and female PD patients; 2 common to both analyses (Yq11.1, 5q34)	[56]
Transcriptomics	Affymetrix GeneChip^Ⓡ^ Human Exon 1.0 ST Array	Peripheral blood leukocytes: 29 female control, 30 female PD	115 DEGs between female PD patients and controls (|FC| > 1.5, *p* < 0.01), enriched for immune function and B cell signaling	[121]
Transcriptomics	Affymetrix GeneChip^Ⓡ^ Human Genome U-133 A Array	Dopaminergic neurons isolated from substantia nigra tissue: 6 male control, 7 male PD, 3 female control, 3 female PD	86 genes differentially expressed in analysis of male control vs. PD and female control vs. PD (FDR < 0.01), 5 of which were also differentially expressed in male control vs. female control	[52]
Transcriptomics	Affymetrix GeneChip^Ⓡ^ Human X3P Array	Dopaminergic neurons isolated from substantia nigra tissue: 4 male control, 4 male PD, 4 female control, 4 female PD	120 genes with sex-specific expression, regardless of PD status (*p* ≤ 0.05) 288 genes differentially expressed in female PD vs. control 292 genes differentially expressed in male PD vs. control	[53]
Epigenomics	Bisulfite pyrosequencing	Platelet mtDNA: 20 male control, 30 male PD, 20 female control, 17 female PD	No sex- or PD-associated mtDNA methylation changes at *MT-TL1*,* MT-CO2 MT-CO2*, or *MT-CO3* genes	[122]
Epigenomics	Illumina Infinium MethylationEPIC BeadChip Array	Neurons isolated from parietal cortex: 30 male control, 33 male PD, 20 female control, 17 female PD	Sex- and PD-specific CpG methylation at *PARK7* (DJ-1), *SLC17A6* (VGLUT2), *PTPRN2* (IA-2β), *NR4A2* (NURR1), and other genes 3 DM CpGs in male PD vs. control analysis, 87 DM CpGs in female PD vs. control analysis	[57]
Epigenomics	Illumina Infinium MethylationEPIC BeadChip Array	Whole blood: 80 male control, 38 male PD, 67 female control, 33 female PD	3 DM regions in male PD vs. control analysis, 69 DM regions in female PD vs. control analysis 70% of models for female DM regions were better explained by inclusion of genotype	[58]

PD: Parkinson’s disease. GWAS: genome-wide association study. FDR: false discovery rate. eQTL: expression quantitative trait locus. r_g_: genetic correlation. *h*^2^: narrow-sense heritability. DEG: differentially expressed gene. FC: fold change. mtDNA: mitochondrial DNA. DM: differentially methylated

Sex differences in gene regulation have been reported for PD risk loci, particularly with respect to transcription (Table 1). For example, the *SRY* gene is expressed from the Y chromosome in males and can regulate dopamine biosynthesis [49]. In cell culture and rodent studies, upregulation of *SRY* is observed following 6-OHDA treatment, suggesting it could play a role in PD susceptibility in males [50, 51]. In addition to the effects of *SRY*, males have higher levels of *SNCA* and *PINK-1* mRNA in dopaminergic neurons and the substantia nigra than females, while females have upregulated expression of neuronal maturation genes [52, 53]. As an increasing number of transcriptomic studies on PD post-mortem brain were published over the past 10–15 years, several groups have recently analyzed publicly available data with respect to male-specific, female-specific, and sex-associated gene expression patterns in the substantia nigra and other brain regions [54–56]. The majority of PD-related differential expression was found in the substantia nigra, and a small number of genes were reported to be differentially expressed within one sex, or in association with sex in two independent substantia nigra meta-analyses: *ARMCX2* (X-linked, upregulated in females and downregulated in males), and autosomal genes *IFITM2* (downregulated in females and upregulated in males), *DYNC1LI1* (downregulated in males), and *REEP1* (downregulated in males) [54, 55]. Of note, three substantia nigra studies also reported a larger proportion of upregulated genes in female PD vs. control comparisons than in male PD vs. control comparisons [52, 53, 56]. While results vary from gene to gene, this suggests a broad trend for decreased overall gene expression and upregulation of PD-specific loci in males with PD and increased overall gene expression in females with PD.

Compared with studies on gene expression, studies on sex-specific epigenetic patterns in PD have been limited thus far. Two transcriptomic meta-analyses reported more histone and chromatin-modifying genes with differential expression in female PD cases than male PD cases [55, 56]. One group also reported sex-dependent DNA methylation patterns in prefrontal cortex neurons isolated from PD patients, and a greater number of DNA methylation alterations in females with PD than males [57]. Finally, we recently found a similar pattern of increased DNA methylation alterations in blood samples of females with PD compared with males [58]. This suggests that while PD is more prevalent in men, epigenetic changes associated with PD may be more widespread in females. Further epigenetic studies of PD in larger populations and multiple tissues will be required to confirm or refute this, supplemented by experimental studies in animal and cell culture models to understand the mechanisms underlying sex-dependent epigenetic regulation in PD. Taken together, current literature suggests that males and females have a similar overall genetic risk for PD, and have differential patterns of transcriptomic and epigenomic regulation, which may contribute to PD susceptibility through unique mechanisms in each sex.

### Intersection of sex, gender, and lifestyle factors

**Fig. 1:**
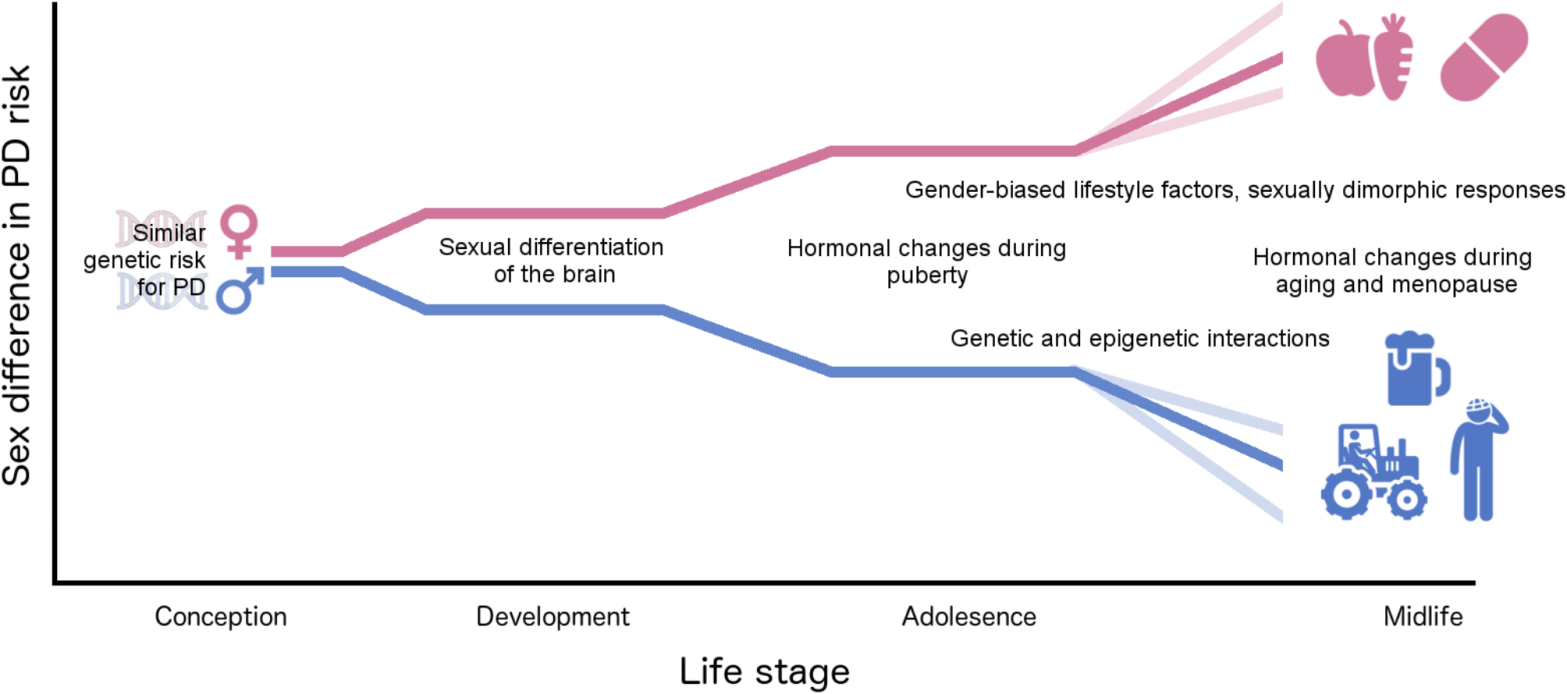
Factors influencing sex differences in Parkinson’s disease risk across the life course. Males and females have a similar genetic risk for PD from conception. Hormonal changes during development, puberty, and aging contribute to sex differences in brain structure, function, and gene regulation. In adolescence and adulthood, gender influences which lifestyle factors contributing to or ameliorating PD risk are encountered, while sex and genetic/epigenetic interactions influence the physiological effects of lifestyle factors and biological embedding of pathways to PD. DNA helix, male and female symbols, food, beverage, pill, tractor, and head injury icons are reproduced from Flaticon with permission [74]

In human population studies of PD, the influence of socially-constructed gender roles and identities are intertwined with sex. Societal gender norms influence the lifestyles women and men lead, and in turn, their risk for developing PD (Fig. 1). One of the best-studied examples of such lifestyle factors that contributes to PD risk and also has a gender bias is exposure to pesticides. Pesticide exposure and farm work are robustly associated with PD in epidemiological studies, and pesticides including rotenone, paraquat, and others cause dopaminergic damage in rodent models of PD [59–61]. In many societies, men are more likely to spray pesticides occupationally and to be exposed to hazardous chemicals in the workplace [62, 63]. This is likely a contributor to the association between pesticide exposure and PD reported in men [64, 65]. However, some regions such as Japan and sub-Saharan Africa have a higher proportion of women working on farms as compared to men working on farms, and do not have the same increased male: female prevalence ratio for PD observed in other areas of the world [61, 66, 67]. Women may also be exposed to pesticides by different routes than men, including gardening and household use, and handling of contaminated objects and clothing [66, 68]. In addition to the gender-based likelihood of exposure, pesticides and neurotoxins interact with biological sex-related factors. Higher levels of adipose tissue in females compared with males increase the risk for pesticide absorption and later release into the blood stream [69]. Changes in estrogen levels during pregnancy, breastfeeding, and menopause also affect susceptibility to negative health effects of pesticide exposure [66, 70]. Finally, pesticide exposures may interact with genetic and epigenetic factors differently in each sex, although this is less well studied [71–73]. In summary, while men traditionally are more likely to experience higher occupational pesticide exposure, this varies depending on geographical region and culture, and exposures through non-occupational means as well as their interactions with biological sex should be considered in the context of risk for PD.

Aside from exposure to pesticides, other lifestyle factors related to PD risk or protection that are typically more common in men include smoking, alcohol consumption, and head injury [75].

Traumatic brain injury (TBI) may increase PD risk through brain inflammation and breakdown of the blood-brain barrier in addition to potential direct neuronal and axonal tissue damage. While the effects of TBI on risk for PD and other neurodegenerative disorders have been well studied in men relative to women due to the higher prevalence of sports- and combat-related accidents for this gender, the interaction between head injury and hormonal status in women and whether this contributes to sex differences in PD and other neurodegenerative disorders is under-studied [76–79]. As female rodents often have improved recovery from TBI paradigms compared with male rodents, and recovery from TBI in human females is affected by menstrual cycle stage, female sex hormones were initially thought to contribute to neuroprotection in TBI [80, 81]. However, a human clinical trial for progesterone in TBI yielded no difference in outcomes, limiting the transferability of these findings [82]. Similarly, although some studies have reported a survival advantage for post-menopausal women after traumatic brain injury compared with men in the same age group, this effect is not observed in all populations [76]. On the whole, it is possible that women may be at lower risk than men for experiencing long-term effects from TBI, including susceptibility to PD; yet, the mechanisms behind this are not fully understood.

In addition to incidence of TBI, alcohol intake is on average higher in men than women, and at moderate (but not low or high) levels, is associated with a decreased risk for PD, possibly through elevation of serum urate [83–86]. Specifically, moderate beer consumption had the strongest association suggesting a protective effect in a meta-analysis of studies on alcohol consumption and PD risk [85]. Several lines of evidence indicate that alcohol intake and/or smoking may reduce PD risk in men to a greater magnitude than in women. First, physiological factors including genetics, hormone levels, and alcohol dehydrogenase activity differ between sexes, with men having a faster ethanol metabolic rate [87]. Second, the interaction between moderate drinking and current smoking status was associated with reduced PD risk for men, but not women, in a Korean population study [88]. Third, smoking was associated with increased β2 nicotinic acetylcholine receptor availability in the striatum of men, and not women, as measured in an American cohort by [^123^I]5-IA SPECT [89]. Finally, the protective effect of smoking against PD was attenuated by post-menopausal hormone use in women participating in the 22-year prospective Nurse’s Healthy Study [90]. Taken together, many lifestyle factors affecting risk for PD are more prevalent in men, and may tip the balance toward or away from PD susceptibility without the protective effects of female sex hormones at play.

On the other hand, some lifestyle factors influencing PD risk are also more common in women or only applicable to women. A 2021 study in a Canadian cohort of PD patients reported that women with PD had higher scores for Mediterranean- and Mediterranean-DASH Intervention for Neurodegenerative Delay (MIND)-like dietary patterns than men with PD [91]. While these dietary patterns were associated with later onset of PD in both genders, the effect size was larger in women, and similar beneficial effects of the MIND diet for PD were reported in an independent majority-female cohort [91, 92]. Further research is required to confirm whether there is a difference in the effectiveness of dietary interventions for PD in men and women, and to understand potential mechanisms. For instance, diet and sex modulate gut microbiota composition, which has been hypothesized to initiate the “body-first” subtype of PD (where gut inflammation associates with formation of alpha-synuclein pathology in the enteric nervous system) [93, 94]. This topic is reviewed further in [93, 94].

Other protective factors against PD that exert different physiological effects in each sex include caffeine intake and physical activity. The inverse association between caffeine intake and risk for PD has a linear dose-response effect in men and a U-shaped effect in women [95]. This U-shaped effect may be due in part to estrogen levels, as individuals with mutant alleles in the estrogen receptor genes *ESR1* and *ESR2* and individuals who do not undergo post-menopausal hormone therapy experience a greater protective effect from caffeine than those undergoing hormone replacement therapy (HRT) or with a normal *ESR1*/*ESR2* genotype [96–98]. Physical activity is also protective against PD in both men and women, though each sex has a distinct physiological response to exercise with respect to lipid metabolism and resting metabolic rate [99–101]. While exercise guidelines have been developed to address sex-specific motor symptoms of PD, such as a focus on progressive multi-directional walking and resistance training for women, the biological underpinnings of the intersections between exercise, sex, and PD are less well studied [102, 103].

On the whole, many exposures and lifestyle factors influencing PD risk have a gender bias, and most will influence disease risk in the context of biological sex-related factors including gonadal hormone levels and gene regulation. This may inform guidance on PD prevention strategies tailored to each sex; for instance, effectiveness of prevention strategies for women will differ based on their menopausal status and whether they are taking hormone therapy. In order to understand PD etiology in men and women, both societal and biological factors should be considered in cohort design, data analysis, and study interpretation.

### Accounting for sex and gender in study design and data analysis

Incorporating sex and gender into PD research begins at the stage of study design. Between 2000 and 2020, US clinical trials in neurology had one of the lowest rates of female representation relative to disability-adjusted life years when compared with trials in other medical fields [104]. For PD in particular, the higher disease prevalence in men and the recruitment of spouses and caregivers as controls may contribute to over-representation of male cases and female controls in research studies [105]. Men with PD are also more likely than women with PD to have a caregiver who can assist with their participation in research and clinical trials, while women with less advanced PD may be caregivers for others in their families [106, 107]. Additionally, participation in lifestyle prevention trials for PD and other conditions differs by gender, suggesting that tailored approaches to increase equity are needed across all aspects of health research including prevention and treatment [104].

Implementing strategies to reduce barriers to research participation and to enhance recruitment of women with PD may help address the gender imbalance in clinical studies. For example, offering remote assessments through video calls and online questionnaires reduces reliance on a caregiver for transportation to the study site [106, 108]. Biological samples such as oral swabs, feces, and small volumes of blood can also be taken using at-home kits, and mailed to the study coordinator, reducing barriers to participate in research [109]. Involving women in design and implementation of PD research studies will likely improve recruitment efforts, and collection of sex- and gender-relevant information such as occupation, lifestyle, and hormonal factors will be key [107]. Finally, policies and guidelines for the inclusion of women in biomedical research have been developed by the World Health Organization, National Institutes of Health, Canadian Institutes of Health Research, and other organizations worldwide (e.g. Asian and African women’s health research initiatives) which provide resources for inclusive study design [110–114]. These guidelines also highlight the importance of including both sexes in animal and in vitro-based experimental models of PD, which have historically been male-biased [115].

While strategies to address sex and gender in PD research should be applied as far upstream as possible, they can also be incorporated at the analysis stage. Cohort sizes can be increased through multi-centre collaborations and/or use of publicly available data, to ensure adequate representation of each sex [116]. If case-control numbers or confounding factors are imbalanced by sex, sub-setting or weighting approaches such as propensity score matching can be applied to reduce the level of bias between groups [117]. Researchers should also consider the role of sex and gender in the molecular etiology of PD when developing hypotheses and analytical approaches. Specifically, adjusting for sex as a covariate will reveal similar effects that are present when averaged across males and females, and might miss sex-dependent effects [13]. On the other hand, stratifying by sex will consider males and females as individual cohorts, and using sex-based interaction models may identify both sex-dependent and sex-independent effects [13]. As sub-setting, stratifying, and interaction analyses require sufficient power, and stratification can sometimes result in spurious detection of sex differences, researchers should ensure adequate sample sizes prior to applying these approaches [118–120].

## Conclusions

In the past decade, progress has been achieved toward elucidating sex differences in the molecular underpinnings of PD and other health conditions. However, as the 2019 Parkinson’s Foundation report on Women and Parkinson’s revealed, many barriers and knowledge gaps still exist in study design, recruitment, and analysis [107]. Given the attention to the inclusion of both men and women in research studies, coupled with our increased capacity to recruit large cohorts, apply new molecular profiling technologies, and integrate multiple data types, scientists have ample and growing opportunities to better characterize PD pathophysiology in both sexes across the life course. In consideration of the intersections between genetics, gene regulation, sex hormones, and lifestyle factors in each sex lies great potential for enhancing equity in PD research and therapeutic development. Ultimately, sex differences are clearly present in PD, and we are edging closer to understanding their complexities.

## Data Availability

No datasets were generated or analysed during the current study.

## Abbreviations

PD: Parkinson’s disease
UPDRS: Unified parkinson’s disease rating scale
OHDA: Hydroxydopamine
GWAS: Genome-wide association study
SNP: Single nucleotide polymorphism
TBI: Traumatic brain injury
MIND: Mediterranean-DASH intervention for neurodegenerative delay
HRT: Hormone replacement therapy
